# A pilot study assessing the addition of a Quit and Win program to pharmacist-led intensive smoking cessation therapy in a predominantly underserved, minority population

**DOI:** 10.18332/tpc/113356

**Published:** 2019-11-26

**Authors:** Kirk E. Evoy, Kentya H. Ford, Sabina 0. Nduaguba, Amber Taylor, Lindsay Thomas

**Affiliations:** 1 College of Pharmacy, The University of Texas at Austin, Austin, Texas, United States; 2 Department of Pharmacy, University Health System, San Antonio, Texas, United States

**Keywords:** smoking cessation, pharmacist, quit and win, financial incentive, underserved, minority

## Abstract

**INTRODUCTION:**

Quit and Win programs (Q&W) have been shown to improve smoking cessation rates by offering potential rewards to encourage smoking cessation. However, few studies have combined Q&W with intensive smoking cessation programs including behavioral counseling and pharmacotherapy, or studied Q&W in underserved, minority populations. This study was conducted to assess the impact on smoking cessation rates of adding a Q&W to intensive smoking cessation therapy in a largely underserved, minority population.

**METHODS:**

This was a single-center, prospective, open-label controlled study. Current smokers received pharmacist-led behavioral counseling and smoking cessation pharmacotherapy. Intervention group patients who successfully quit (verified by self-report and exhaled carbon monoxide) at 1 month and 3 months post-quit date were entered into a draw for $1000. The control group received the same smoking cessation services, but without a monetary incentive.

**RESULTS:**

Enrollment was 111 patients (N=85 in the intervention group), made up of predominantly underserved (82% had annual household income <$25000), minority (69.1%), and female (58%) patients. Groups were similar except the intervention group had lower educational and income levels, while the control group was more likely to smoke more than 1 pack per day. Quit rates at 3 months were 27% and 19% in the intervention and control groups, respectively (p=0.22). Female gender (OR=2.84; p=0.04) and Fagerström score (OR=0.71; p<0.01) were significant predictors of quitting.

**CONCLUSIONS:**

The addition of Q&W to intensive smoking cessation services increased clinic referrals and numerically improved cessation rates, although this difference was not statistically significant, possibly due to high attrition of the study.

## INTRODUCTION

Tobacco use is the leading known preventable cause of death and a major contributor to four of the five leading causes of death in the United States: heart disease, cancer, respiratory disease, and cerebrovascular disease^1^. Furthermore, associated negative health consequences substantially increase economic burden on the healthcare system^2,3^. Efforts targeting smoking cessation have the potential to decrease morbidity, mortality, and the economic burden associated with smoking and secondhand smoke exposure^4^. However, a 2016 study found that a lower proportion of underserved smokers in Texas used smoking cessation medications (1%) than in any other state in the United States (US)^5^. Thus, providing incentives to seek smoking cessation treatment in these populations might prove especially beneficial.

Quit and Win programs (Q&W) are an example of one such effort to encourage smoking cessation. Q&W is an initiative in which enrolled smokers who successfully quit smoking for a pre-specified period of time enter into a draw to win a prize (e.g. money, vacations, consumer goods etc.)^6^. The prize serves as additional incentive for smokers to initiate and maintain quit attempts. The success of Q&W has been documented in numerous studies, dating back to the 1980s when the Minnesota Heart Health Program organized the first Q&W. Since then, numerous Q&W have been conducted and have demonstrated an 8–20% increase in quit rates at 3 months compared to offering no incentive^7-12^. This represents a substantial improvement, considering that typical treatment with pharmacotherapy and behavioral counseling generally produces less than 30% success rates, even in clinical trial settings^13^.

Although a number of previous Q&W studies have been reported in the literature, few have included a control group or have been conducted in a pharmacist-led clinic^7^. Furthermore, few studies have assessed the efficacy of Q&W in addition to intensive smoking cessation therapy (i.e. face-to-face counseling plus pharmacotherapy), and few have been conducted with underserved or minority populations. To our knowledge, this pilot study is the first controlled study to estimate the extent to which the addition of a Q&W to pharmacist-led behavioral counseling and pharmacotherapy (intervention) improves the odds of smoking cessation compared to behavioral counseling and pharmacotherapy alone (control) in an underserved, largely minority population.

## METHODS

### Study design

This single-center, prospective, open-label controlled study conducted at a primary care clinic in San Antonio, Texas, integrated a Q&W into an established, pharmacist-led intensive smoking cessation service. The study was approved by The University of Texas Health San Antonio Institutional Review Board (Protocol HSC20160393H).

### Recruitment and enrollment

A convenience sample was used for the study, as recruitment was driven by provider referral to the service. Patients who were current smokers were referred to the on-site pharmacist-led smoking cessation program at the discretion of their primary care provider. Patient recruitment flyers were posted throughout the clinic, and providers within the clinic were made aware of the Q&W to increase referrals. The demographic composition of this clinic was predominantly underserved, minority patients.

Inclusion criteria were: age ≥18 years, provision of informed consent, current smoking status (patient reported current use of inhaled carbon monoxide (CO) producing tobacco products, including cigarettes, cigars, or pipes, confirmed by exhaled CO level ≥10 ppm), ready to quit smoking in the next 30 days, and agreement to return to the clinic for both follow-up visits at 1 month and at 3 months. Patients referred to the smoking cessation service who met inclusion criteria received verbal and written explanation of the study and were offered voluntary enrollment during the initial visit. Written informed consent was obtained prior to enrollment.

Study group assignment was open-label based on date of study enrollment, with intervention and control groups recruited and treated non-concurrently. Patients attending their first visit to the pharmacist service from 1 March to 31 August 2017 were included in the intervention group, whereas patients enrolled between 1 September to 30 November 2017 were included in the control group. This uneven recruitment window for the two groups was established prior to beginning study enrollment based on the limited time frame in which grant funds were available and in order to maximize the number of participants with access to the intervention, while still enrolling a control group.

To encourage enrollment and return for follow-up visits, participants in both groups were offered $10 per visit for attending the visits at 1 month and at 3 months follow-up and an additional $10 for attending both (up to $30 total). Additional measures taken to improve study follow-up rates included reminder telephone calls prior to the appointment and after missed appointments, in an attempt to reschedule visits.

### Standard care

Standard care provided to all patients included: a) one-on-one behavioral counseling using motivational interviewing to assist in preparing for the quit attempt and overcoming barriers to cessation, and b) smoking cessation pharmacotherapy. This smoking cessation service was led by one pharmacist and was provided free to patients. The pharmacist had prescription authority under a collaborative practice agreement.

The face-to-face visits lasted approximately 20–30 minutes. Patients were encouraged to attend at least 3 to 4 in-person visits, with further follow-up available based on the patient’s desire for additional assistance. Short telephone follow-up appointments were also available based on patients’ preference, although patients were required to attend in person the baseline appointment, and the follow-up visits at 1 month and at 3 months. Patients were given a 2-week window around their follow-up dates to complete their required study.

Medication choice (nicotine replacement therapy NRT should be within brackets here, i.e., nicotine replacement therapy [NRT], varenicline, bupropion, or a combination of these medications) was individualized based on patient comorbidities, previous patient experience with smoking cessation pharmacotherapy, and patient preference. The pharmacist assessed which options were appropriate for the individual patient and described to the patient the advantages and disadvantages of each option. Choice of medication was largely based on patient preference following this discussion. If a patient was prescribed medication to aid their quit attempt, they additionally received focused medication counseling and follow-up. Medications were not provided at no cost through the study; patients were required to pay their insurance co-payment (if applicable) or cash price for the medication.

### Intervention and control conditions

The intervention studied was the addition of a Q&W to the standard care pharmacist-led intensive smoking cessation service described above. Patients in the intervention group were informed at the outset of the study that if they returned for the follow-up visits, at 1 month and at 3 months, and had quit smoking at both time points, as indicated by both self-report and exhaled CO, they would be eligible to win a $1000 prize awarded to one study participant. Intervention group patients eligible for the contest draw were assigned sequential numbers. The winner was determined via a computer-based random number generator.

A control group was also studied, but during a period in which the Q&W was not offered. Patients enrolled in the pharmacist-led smoking cessation service, within the 3 months directly after recruitment for the Q&W had ended, were recruited to participate in the study as a member of the control group. Participants in the control group were provided the same standard care intensive smoking cessation service described above, but without the opportunity to enter the $1000 prize draw.

### Measures and data collection

Baseline demographics (age, gender, race, education, income, marital status, payer status, and co-morbidities) and questions addressing smoking history: type of tobacco product(s) used, number of past quit attempts, past use of smoking cessation pharmacotherapy, past receipt of behavioral counseling for smoking cessation, amount of tobacco used per day, stage of readiness to quit, and level of nicotine dependence using the Fagerström test for nicotine dependence, were collected during initial patient interview. Smoking status was assessed at baseline, at 1 month, and at 3 months follow-up appointments. The questions ‘Do you currently smoke cigarettes, cigars, or pipes?’ and ‘Have you smoked cigarettes, cigars, or pipes in the last seven days?’ were used to determine current use and 7-day point prevalence, respectively. To objectively measure smoking status, the patient’s exhaled CO level was obtained using the Smokerlyzer^®^ CO monitor (CoVita/Bedfont, Santa Barbara, CA). A CO reading ≥10 ppm indicated current smoker status. Additionally, at the follow-up at 3 months, patients were asked whether the Q&W that was provided had increased their motivation to quit.

### Statistical analyses

Primary analysis was performed using the intention-to-treat approach, for which individuals who did not attend the follow-up visits were considered to have had unsuccessful quit attempts at those time points. A secondary per-protocol analysis was also performed that included only those patients completing both follow-up visits at 1 month and at 3 months. The primary endpoint was confirmed quit status as defined by both self-reported smoking cessation and CO level <10 ppm at 3 months. Two-sample t-tests (continuous variables), χ^2^ tests of independence (nominal variables), and Kruskal-Wallis (ordinal variables) tests were used to compare baseline characteristics between control and intervention groups. For nominal variables with bivariate tables having 25% or more cells with expected size less than five, the cells were collapsed and the chi-squared results compared with the original table. Where similar, the uncollapsed frequencies were reported. Fisher’s exact test was applied for nominal variables with only two levels that had at least one expected cell size of less than five. Logistic regression and generalized estimating equation (GEE) were used to compare quit rate between intervention arms and to evaluate predictors of quit status. The following variables were tested in univariate analyses as potential predictors of quit status at p<0.1: age, gender, race/ethnicity, marital status, education, income, Fagerström test, number of physical comorbidities, and number of mental comorbidities. Only factors within this threshold were included in the final regression models. Significance level was set a *priori* at 0.05. It was estimated that a sample size of 132 participants would be needed to detect a difference of 23% between the intervention and control groups at 3 months (α=0.05, β=0.8). This difference was chosen based on the observed improvement in cessation rates in participants of a Q&W contest versus control patients in a prior study^8^.

**Table 1:** Descriptive characteristics of study participants

	*Total (N=111)*	*Intervention (N=85)*			*Control (N=26)*	*p*
**Age** (years), mean (SD)^a^	52.2 (10.3)	52.8 (10.0)			50.2 (11.1)	0.26
**Gender**, n (%)^b^						
Male	47 (42.3)	37 (43.5)			10 (38.5)	0.65
Female	64 (57.7)	48 (56.5)			16 (61.5)	
**Race/Ethnicity**, n (%)^b^						
Hispanic	46 (41.8)	38 (45.2)			8 (30.8)	0.35
Caucasian	34 (30.9)	24 (28.6)			10 (38.5)	
Black	27 (24.6)	20 (23.8)			7 (26.9)	
Asian/Hawaiian/Pacific Islander/Other	3 (2.7)		2 (0)		1 (3.8)	
Missing	1 (0.9)		1 (1.2)		0	
**Marital Status,** n (%)^b^						
Married	33 (29.7)		22 (25.9)		11 (42.3)	0.32
Divorced	24 (21.6)		21 (24.7)		3 (11.5)	
Single	42 (37.8)		33 (38.8)		9 (34.6)	
Other	12 (10.8)		9 (10.6)		3 (11.5)	
**Education**, n (%)^c^						
Some school, no diploma	44 (39.6)		38 (44.7)		6 (23.1)	0.05
High school diploma	29 (26.1)		20 (23.5)		9 (34.6)	
Some college	25 (22.5)		20 (23.5)		5 (19.2)	
College degree/graduate degree	13 (11.7)		7 (8.2)		6 (23.1)	
**Income** (US$), n (%)^c^						
<25000	91 (82.0)		73 (85.9)		18 (69.2)	0.04
25000–50000	16 (14.4)		11 (12.9)		5 (19.2)	
50001–75000	1 (0.9)		0 (0.0)		1 (3.9)	
>75000	3 (2.7)		1 (1.2)		2 (7.7)	
**Health insurance,** n (%)^b^						
Carelink^d^	56 (50.5)		42 (49.4)		14 (53.9)	0.18
Medicare	12 (10.8)		11 (12.9)		1 (3.9)	
Medicaid	27 (24.3)	23 (27.1)			4 (15.4)	
Medicare + Medicaid	6 (5.4)	3 (3.5)			3 (11.5)	
Private	9 (8.1)	5 (5.9)			4 (15.4)	
None	1 (0.9)	1 (1.2)			0 (0)	
**Tobacco use,** n (%)^e^						
Single dosage form of tobacco used	96 (86.5)	71 (83.5)			25 (96.2)	0.019
Multiple forms of tobacco used	15 (13.5)	14 (16.5)			1 (3.8)	
Amount* of tobacco used, n (%)^c^						
1–5	10 (9.0)	10 (11.8)			0 (0.0)	0.045
6–10	25 (22.5)	20 (23.5)			5 (19.2)	
11–20	62 (55.9)	46 (52.1)			16 (61.5)	
21–30	11 (9.9)	9 (10.6)			2 (7.7)	
31–40	3 (2.7)	0 (0.0)			3 (11.5)	
**Packs per day,** n (%)^e^						
≤1	97 (87.4)		76 (89.4)	21 (80.8)		0.31
>1	14 (12.6)		9 (10.6)	5 (19.2)		
**CO level at baseline,** median (IQR)^c^	25 (18–36)		25 (18–35)	27 (19–37)		0.72
**Fagerström score at baseline,** mean (SD)^a^	4.79 (2.03)		4.62 (2.01)	5.35 (2.04)		0.11
**Fagerström score dependence level at baseline,** n (%)^c^						
Low (1–2)	22 (19.8)		19 (22.4)	3 (11.5)		0.31
Low to Moderate (3–4)	24 (21.6)		18 (21.2)	6 (23.1)		
Moderate (5–7)	58 (52.3)		43 (50.6)	15 (57.7)		
High (≥28)	7 (6.3)		5 (5.9)	2 (7.7)		
**Number of prior quit attempts,** median (IQR)^c^	3 (1–4)		3 (1–4)	3 (2–5)		0.09
**Tried pharmacotherapy prior to study,** n (%)^e^						
No	48 (43.2)		36 (42.4)	12 (46.2)		0.73
Yes	63 (56.8)		49 (57.6)	14 (53.8)		
**Pharmacotherapy tried prior to study,** n (%)						
NRT^b^	54 (48.7)		42 (49.4)	12 (46.2)		0.77
Chantix^e^	17 (15.3)		13 (15.3)	4 (15.4)		0.99
Bupropion^e^	9 (8.1)		7 (8.2)	2 (7.7)		0.99
E-cigarette^e^	3 (2.7)		3 (3.5)	0 (0.0)		0.99
Behavioral counseling^e^	14 (12.6)		11 (12.9)	3 (11.5)		0.99

a t-test.

b chi-squared test.

c Kruskal-Wallis test.

d Carelink is a county-subsidized payment plan for low-income residents of Bexar County – this is not insurance but typically serves as the alternative to prescription insurance for most Carelink members.

e Fisher’s test. SD: standard deviation, CO: carbon monoxide, IQR: interquartile range, NRT: nicotine replacement therapy.

* Cigarettes, cigars and pipes.

## RESULTS

### Intent-to-treat analysis

#### Description of participants

A total of 111 patients participated in the study, 85 (76.6%) in the intervention group and 26 (23.4%) in the control group. Table 1 gives the baseline characteristics of the study participants. The mean age was 52.2 years (standard deviation, SD=10.3 years). The majority of participants were female (57.7%), Hispanic (41.8%), single (37.8%), had a high school education or less (65.7%), and earned less than $25000 annually (82.0%). Less than 4% of patients had a household income of $50000 or greater, and 74.8% had Medicaid insurance or Carelink (a subsidized healthcare payment plan available to underserved patients in Bexar County, Texas, which serves as a health insurance alternative for a subset of underserved patients in San Antonio). The average Fagerström score at baseline was 4.8 (SD=2) with most participants (52.3%) having moderate nicotine dependence (Table 1). There were no significant differences between the intervention and control groups in nicotine dependence level at baseline (Table 1) or the type of cessation aid used in the study (Tables 2). Baseline characteristics that significantly differed between the intervention and control groups at baseline were level of education, income, and amount of tobacco used per day.

**Table 2:** Medications prescribed per group and quit rates at 1 month and at 3 months

*Measure of Quit Rate*	*Total (N=111)*	*Intervention (N=85)*	*Control (N=26)*	*p*
**Pharmacotherapy prescribed during study,** n (%)^a^				
NRT	76 (72)	59 (72)	17 (71)	0.91
Chantix or bupropion	30 (28)	23 (28)	7 (29)	
No medication used (n)	5	3	2	
**Quit rate at 1 month,** n (%)^a^				
**Current smoker**				
Yes	79 (71)	60 (71)	19 (73)	0.81
No	32 (29)	25 (29)	7 (27)	
**Smoked in past 7 days**				
Yes	80 (72)	60 (71)	20 (77)	0.53
No	31 (28)	25 (29)	6 (23)	
**CO level (ppm) for smoking status**					
≥10	81 (73)	60 (71)	21 (81)	0.31
<10	30 (27)	25 (29)	5 (19)	
**CO smoking status + self-report**				
Still smoking	81 (73)	60 (71)	21 (81)	0.31
Not smoking	30 (27)	25 (29)	5 (19)	
**Quit rate at 3 months,** n (%)^a^				
**Current smoker**				
Yes	79 (71)	60 (71)	19 (73)	0.81
No	32 (29)	25 (29)	7 (27)	
**Smoked in past 7 days**				
Yes	80 (72)	60 (71)	20 (77)	0.53
No	31 (28)	25 (29)	6 (23)	
**CO smoking status**				
Current smoker (CO ≥10 ppm)	79 (71)	58 (68)	21 (81)	0.22
Not smoking (CO <10 ppm)	32 (29)	27 (32)	5 (19)	
**CO smoking status + self-report**					
Still smoking	83 (75)	62 (73)	21 (81)	0.42
Not smoking	28 (25)	23 (27)	5 (19)	

a Chi-squared test. NRT: nicotine replacement therapy, CO: carbon monoxide; ppm: parts per million.

Study participants had an average of three comorbidities (Supplementary file), with 58.6% having seven or more comorbidities. There were no significant differences in number of comorbidities between intervention and control groups.

Nearly all patients (95.5%) used one or more pharmacotherapy agents for quitting smoking (Table 1). One patient quit without medication and was not included in the test of comparisons of pharmacotherapy. Two participants in the intervention group were prescribed both NRT and bupropion and were classified as having received prescription cessation aid. Of those who received cessation aids during the study, 71.7% received over-the-counter NRT, and 28.3% received prescription agents.

#### Quit rate and predictors of quit rate

The quit rate at 1 month was 29% in the intervention group versus 19% in the control group (p=0.31) (Table 2). Quit rate at 3 months was 27% in the intervention group and 19% in the control group (p=0.42). Race/ethnicity, income, and number of packs per day, which have been suggested by some studies to impact on Q&W success, were not significant predictors of quit rate (Supplementary file)^7^. In the unadjusted models, only gender and Fagerström score were significantly associated with quitting. These two variables were entered into the final model examining the association between treatment assignment and quit rate (Table 3). There were no statistically significant differences in quit rate between the intervention and control group after controlling for gender and Fagerström scores (odds ratio, OR=1.38; 95% CI: 0.45–4.19; p=0.57). Also, gender and Fagerström scores remained significant predictors of quit rate in the overall adjusted model. The odds of quitting were 2.62 times higher in females compared to males (OR=2.62; 95% CI: 1.04–6.63; p=0.04). For every unit increase in Fagerström score, the odds of quitting decreased by 28% (OR=0.72; 95% CI: 0.57–0.89; p<0.01). Additional analysis was conducted for Hispanics only. There was no significant difference in quit rates between Hispanics assigned to the treatment group versus control group at 1 month (29% vs 25%; OR=1.22; 95% CI: 0.21–7.01; p=0.82) or at 3 months (26% vs 25%; OR=1.07; 95% CI: 0.19–6.20; p=0.94).

**Table 3:** Adjusted models of the association between quit rate (CO smoking status plus currently smoking) and treatment assignment

	*Overall Model^a^ OR (95% CI), p*	*At 1 Month^b^ OR (95% CI), p*	*At 3 Months^b^ OR (95% CI), p*
**Treatment group** (Ref. Control)	1.38 (0.45–4.19), 0.57	1.48 (0.48–4.55), 0.49	1.24 (0.39–3.92), 0.71
**Gender** (Ref. Male)	2.62 (1.04–6.63), 0.04	2.25 (0.89–5.70), 0.09	3.25 (1.18–9.01), 0.02
**Fagerström score**	0.72 (0.57–0.89), <0.01	0.75 (0.59–0.94), 0.01	0.69 (0.53–0.88), <0.01

a Generalized estimating equation controlling for gender and Fagerström test score.

b Binomial logistic regression controlling for gender and Fagerström test. CO: carbon monoxide, OR: odds ratio, CI: confidence interval.

### Per-protocol analysis

Twenty patients (23.5%) in the intervention group and six patients (23.1%) in the control group completed both follow-up visits at 1 month and at 3 months. There was no significant difference between the intervention and control groups in the proportion of participants who completed the study. Compared to those who did not complete the study, those who completed the study were more likely to have a high school diploma or higher level of education (76.9% vs 55.3%; p=0.04), more likely to earn over $25000 (34.6% vs 12.9%; p=0.02), more likely to have tried a cessation aid prior to the study (76.9% vs 50.6%; p=0.02), and less likely to have a moderate or high level of nicotine dependence (34.6% vs 65.8%; p=0.01).

Among those who completed the study, participants in the intervention group were more likely than control participants to have tried cessation aids in the past (90% vs 33.3%; p=0.01). CO level at 1 month was significantly lower in the control group [median (IQR): 1.5 (1–2) vs 3 (2–5); p=0.04], but not significantly different at 3 months. Group assignment was not significantly associated with quit rate (OR=0.17; 95% CI: 0.01–2.45; p=0.19). Gender was the only significant predictor of quit rate in the per-protocol analysis. The odds of quitting tobacco use were 8.85 times higher in females compared to males (OR=8.85; 95% CI: 1.13–69.56; p=0.04). Full details of the per-protocol analysis can be requested from the first author (KE).

## DISCUSSION

Among 111 participants in the present Q&W pilot study, 27% of patients in the intervention group (N=85) were abstinent at the 3 months follow-up, as confirmed by both self-report and CO verification, compared with 19% (p=0.42) of patients in the control group (N=26). Q&W programs are not a new concept for encouraging smoking cessation, although few have been conducted as controlled studies. A Cochrane Review identified five Q&W studies, similar to our study, that included a non-contest control group^7^. Three of the five studies did identify significant improvement with quit rates in the Q&W group ranging 8–20% higher than the control. However, the Cochrane Review noted that although the controlled studies suggest that there was an increase in quit rates among participants compared to control populations, firm conclusions could not be drawn from the available studies given the limited number of controlled studies conducted, as well as the small sample sizes and methodological limitations. Given limited research, there remains a need for controlled studies such as the one described here to provide additional evidence regarding the effectiveness of this intervention. Our study builds upon prior research, uniquely focusing on the effectiveness of a Q&W program combined with intensive smoking cessation therapy in an underserved, largely minority population.

Among the studies included in the Cochrane Review, intervention group quit rates ranged from 7.3% to 27.0% while control group quit rates ranged from 0.6% to 10.0%^7^. However, the studies in the Cochrane Review measure continued abstinence at 6 months and at 12 months, whereas our trial was of shorter duration, measuring abstinence at 1 month and at 3 months. Thus, abstinence rates might have diminished to some extent for the longer follow-up.

Unlike most prior Q&W, the present Q&W was offered in addition to intensive smoking cessation therapy (i.e. one-on-one patient counseling plus pharmacotherapy). Past studies have generally provided the Q&W alone or with minimally intensive smoking cessation services (e.g. educational brochures, opportunities to call into a tobacco cessation quitline etc.)^7^. Only one controlled study, conducted by Hawk et al.^14^, combined the Q&W with pharmacotherapy. In that study, participants were offered vouchers for free NRT. However, this was still considered minimally intensive given that proactive counseling was not offered. Furthermore, unlike the present study, the only pharmacotherapy available was NRT. Similar to the present study, Hawk et al.^14^ predicted a higher rate of success when combining a Q&W with pharmacotherapy, but they did not observe a difference. The per cent abstinence (self-reported 7-day point prevalence) was 26% in the NRT-only group and 27% in the Q&W + NRT group. When the participants were asked about the importance of NRT samples, more than 60% marked it as ‘very important’. Although free medication was not provided in the present study, one-on-one counseling and prescriptions for pharmacotherapy were available to all study participants. This methodology led to a considerably higher quit rate among our control group than was seen in previous studies.

Compared to past studies in which the control groups had low quit rates, the higher quit rate in the control group of the present study likely reduced the odds of observing a statistically significant difference between groups. Because no controlled trial, combining Q&W with intensive pharmacotherapy, was identified, past studies might not have provided a reasonable estimation of the between-group differences expected. For our power calculation, we estimated the effect size based on a study by Hahn et al.^8^. This study was chosen because it was among the most methodologically sound Q&W studies published, and one in which the authors reported cessation rates at 3 months. However, the Hahn et al.^8^ study did not incorporate intensive smoking cessation therapy alongside the Q&W. As a result, it is likely that the between-group difference observed in the present study was attenuated given the higher than expected quit rates in the control group. Furthermore, recruitment was slower than anticipated during the study period and our goal sample size was not met. This resulted in the present study being underpowered to detect a difference.

Moreover, attrition rates in the present study were significantly higher than expected. Based on previous study results, an attrition rate of approximately 20% at 3 months was estimated^8^. However, despite measures taken to improve study follow-up rates (e.g. providing payment for attendance at the 1 month and at the 3 months study visits and placing phone calls to participants prior to their appointment to remind them, as well as after missed appointments to attempt to reschedule a visit), only 32.4% of participants completed the follow-up at 3 months, and only 23.4% of participants completed all study visits. There are several potential reasons for this high attrition rate, one being that the study was conducted in a largely underserved population in which participants might lack transportation for appointments or working telephone numbers for reminder calls. Participants who did not complete the study, were more likely not to have completed high school (44.7% vs 23.1%; p=0.04), have earned less than $25000 (87.1% vs 65.4%; p=0.02), have had higher numbers of psychiatric comorbidities (1 vs 0.5; p=0.02), have had higher baseline Fagerström scores (5.02 vs 4.04; p=0.03), and have previously not used medications to try to quit smoking (49.4% vs 23.1%; p=0.02). It also appears that in both groups, those who were successful in their quit attempt were much more likely to complete the study as the quit rates were significantly higher in both groups in the per-protocol analysis. Given that patients who did not attend the visit were presumed to be still smoking, this high study attrition reduced the ability to detect a significant difference between groups.

Another unique feature of the present study was the patient population recruited. As all patients were recruited from the same clinic in Southeast San Antonio, Texas, the convenience sample included a large proportion of minority (69%), particularly Hispanic (41.8%), and underserved patients (82% had a household income of <$25000 per year). Additionally, for the majority of patients (66%), the highest level of education attained was high school education or less. These demographics are distinct from those of most Q&W studies^7^. Of the controlled Q&W studies included in the Cochrane Review, only one (Hawk et al.^14^) achieved a significant minority population by targeting minorities in their recruitment strategies. Similarly, only one study (Hahn et al.^8^) included significant numbers of low-income participants. Hawk et al.^14^ observed no difference in quit rates between non-Hispanic Whites (27%) and non-Whites (28%; 95% CI: 0.67–1.69)^14^. Hahn et al.^8^ reported that women, ethnic minorities, and those with an income below $25000 were as likely to quit as men, Whites, and those with a higher income. In our study, neither race/ethnicity nor income or education level were significant predictors of treatment success. This is important because smoking is generally more often linked to lower educational level, and low income smokers have been identified as having higher risk of smoking-related diseases and lower likelihood to be reached by most tobacco cessation approaches^15^. Furthermore, studies have identified that participants in Q&W contests tend to be more highly educated than smokers in the general population, and that higher education status is associated with higher rates of achieving tobacco cessation^7^. Finally, the present study included a higher proportion of females and identified that females were 2.64 times more likely to quit compared to males. Previous studies have typically shown females to be less or equally likely to be successful in smoking cessation^16^.

Results from past studies show that Q&W typically result in significant increases in the proportion of smokers making quit attempts within a community^7^. Thus, it has been proposed that the public health impact (i.e. cessation rate x participation rate) might be the best measure of the impact of the program, and often shows a greater impact when comparing the results of the intervention versus the control than simply looking at the cessation rate alone. Indeed, in the present study, the Q&W appears to have increased patient motivation to make a quit attempt. In the clinic in which this study was conducted, smoking cessation referrals to the pharmacist-led smoking cessation service more than doubled during the intervention phase (N=85) compared to the number (N=42) over the same six-month period the prior year (i.e. March–September 2016). Additionally, the 14 patients enrolled per month during the intervention phase of the study represented a much higher rate than the 9 patients enrolled per month during the control group recruitment period. Furthermore, 44% of patients in the intervention group reported that the contest encouraged them to make a quit attempt. Two of the studies included in the Cochrane review^9,17^ reported the majority of participants claimed the financial incentive did not motivate them to enter the contest.

### Limitations and strengths

This study has some limitations. As previously mentioned, we were unable to recruit the goal sample size during the study period, and had higher than expected attrition. Furthermore, it appears that the 23% difference in quit rate predicted for our intervention and control groups was too high, likely based on the fact that our control group also received intensive smoking cessation therapy and that we used the stricter primary outcome criteria of exhaled CO confirmed quit rate, rather than simply patient-reported quit rates. As a result, the present study was underpowered to show a significant difference between groups, despite the numerically higher quit rate in the intervention group. Another limitation is the open-label design and non-concurrent enrollment of study groups. While randomization of participants would have been ideal, the single-center design and clinic logistics would make this difficult for a number of reasons. First, during the intervention phase, the Q&W contest was advertised throughout the clinic; therefore, the results of those randomized to the control group could be biased through knowing that they did not qualify for the prize draw. Furthermore, because the intervention entails providing a Q&W as motivation and assessing whether this additional motivation improves cessation rates, group assignments could not be concealed from the patient after randomization. Another limitation is that continued abstinence beyond the duration of the contest was not measured. Follow-up at 6 months and at 12 months, used in some past Q&W studies, would have been preferred but was not feasible given the timeline of the funding source. It is possible that some patients quit in hope of winning the prize but do not remain abstinent in the long-term. Finally, the fact that this study included patients actively recruited within a single clinic, and that all patients were treated by the same pharmacist, limits the generalizability of our findings. However, this design does increase the internal validity of the findings by ensuring the care provided was uniform among all patients. Additional strengths include comprehensive data collection and use of both subjective and objective measures (e.g. CO level) to validate non-smoking status. Only one other study in the Cochrane Review of controlled Q&W studies incorporated biochemical validation, as was done here. The present study also contributes to the overall body of smoking cessation literature by assessing an intervention that has been shown effective (Q&W) in a unique patient population made up primarily of underserved, minority patients. Furthermore, few previous studies added a Q&W to intensive smoking cessation therapy, and no previous study has specifically assessed the addition of a Q&W to a pharmacist-led smoking cessation clinic.

## CONCLUSIONS

This pilot study demonstrated the feasibility of conducting a Q&W contest alongside a previously established, pharmacist-led smoking cessation service, within an underserved, minority population. A statistically significant improvement in quit rate was not observed for those enrolled in the Q&W. However, given that the Q&W was successful in increasing the number of patients trying to quit, and those participating in the program did achieve numerically higher smoking cessation rates, further study is warranted to further evaluate the efficacy of adding Q&W interventions to intensive smoking cessation therapy. To increase the likelihood of achieving appropriate power in subsequent studies, a smaller estimated effect size should be used, additional measures to decrease attrition rates should be implemented and larger numbers of patients should be recruited.
