# Supplementary material for: A pilot study assessing the addition of a Quit and Win program to pharmacist-led intensive smoking cessation therapy in a predominantly underserved, minority population
Source: Tob Prev Cessat. 2019 Nov 26;5:44. doi: 10.18332/tpc/113356 (PMC7205166; doi:10.18332/tpc/113356)
Supplement: Supplementary file 1 [file TPC-5-44-s1.pdf]

SUPPLEMENTARY APPENDIX A: Per-Protocol Analysis

Appendix A1: Descriptive Characteristics of Study Participants (Per-protocol analysis)

	Total (N=26)	Intervention (N=20)	Control (N=6)	*p*-value
Age, years (Mean, SD)[a]	54.6 (11.0)	57.3 (7.3)	45.5 (16.6)	0.14
Gender (N, %)[b]				
Male	10 (38.5)	7 (35.0)	3 (50.0)	0.64
Female	16 (61.5)	13 (65.0)	3 (50.0)	
Race/Ethnicity (N, %)[c,e]				
Hispanic	8 (32.0)	5 (26.3)	3 (50.0)	0.54
Other minorities	7 (28.0)	6 (31.6)	1 (16.7)	
Caucasian	10 (40.0)	8 (42.1)	2 (33.3)	
Missing	1	1	0	
Marital status (N, %)[c,e]				
Married	7 (26.9)	4 (20.0)	3 (50.0)	0.25
Divorced	5 (19.2)	5 (25.0)	0 (0)	
Single	12 (46.2)	10 (50.0)	2 (33.3)	
Other	2 (7.7)	1 (5.0)	1 (16.7)	
Education (N, %)[d]				
Some school, no diploma	6 (23.1)	5 (25.0)	1 (16.7)	0.55
High school diploma	8 (30.8)	6 (30.0)	2 (33.3)	

Some college	7 (26.9)	6 (30.0)	1 (16.7)	
College degree	5 (19.2)	3 (15.0)	2 (33.3)	
Graduate degree	0 (0)	0 (0)	0 (0)	
Income (N, %)[d]				
<$25,000	17 (65.4)	14 (70.0)	3 (50.0)	0.27
$25,000-$50,000	8 (30.8)	6 (30.0)	2 (33.3)	
$50,001-$75,000	1 (3.8)	0 (0)	1 (16.7)	
>$75,000	0 (0)	0 (0)	0 (0)	
Amount of tobacco used[d]				
0-5	1 (3.8)	1 (5.0)	0 (0)	0.88
6-10	8 (30.8)	6 (30.0)	2 (33.3)	
11-20	17 (65.4)	13 (65.0)	4 (66.7)	
21-30	0 (0)	0 (0)	0 (0)	
31-40	0 (0)	0 (0)	0 (0)	
Number of quit attempts (Median, IQR)[d]	3 (1-4)	2.5 (1-4)	4 (3-4)	0.10
Tried cessation aid prior to study (N, %)[b]				
No	6 (23.1)	2 (10.0)	4 (66.7)	0.10
Yes	20 (76.9)	18 (90.0)	2 (33.3)	
Cessation aids tried prior to study (N, %)				
NRT	18 (69.2)	17 (85.0)	1 (16.7)	
Chantix	6 (23.1)	6 (30.0)	0 (0)	

Bupropion	3 (11.5)	2 (10.0)	1 (16.7)	
E-cig	0 (0)	0 (0)	0 (0)	
Behavioral counseling	7 (26.9)	7 (35.0)	0 (0)	

[a]t-test; [b]Fisher's test; [c]Chi-squared test; [d]Kruskal-Wallis test; [e]Validity of Chi-square test questionable due to small cell sizes. Abbreviations: SD = standard deviation; IQR = interquartile range; NRT = nicotine replacement therapy.

SUPPLEMENTARY APPENDIX A2: Physical and Psychiatric Comorbidities among Study Participants (Per-protocol analysis)

	Total (N=26)	Intervention (N=20)	Control (N=6)	*p*-value
Number of comorbidities (Median, IQR)				
Overall[a]	2.5 (1-3)	3 (1.5-3)	1.5 (0-2)	0.04
Physical[a]	1 (1-3)	2 (1-3)	0.5 (0-1)	0.048
Psychiatric[a]	0.5 (0-1)	0.5 (0-1)	0.5 (0-1)	0.76
Number of comorbidities (N, %)[b]				
<3 comorbidities	13 (50.0)	8 (40.0)	5 (83.3)	0.16
≥3 comorbidities	13 (50.0)	12 (60.0)	1 (16.7)	
≥1 comorbidity (N, %)				
Overall[b]	24 (92.3)	20 (100)	4 (66.7)	0.046
Physical[b]	21 (80.1)	18 (90.0)	3 (50.0)	0.06
Psychiatric[b]	13 (50.0)	10 (50.0)	3 (50.0)	1

Physical Comorbidities				
COPD	6 (23.1)	6 (30.0)	0 (0)	
Asthma	0 (0)	0 (0)	0 (0)	
HTN	15 (57.7)	12 (60.0)	3 (50.0)	
HLD	12 (46.2)	11 (55.0)	1 (16.7)	
CHF	0 (0)	0 (0)	0 (0)	
MI/stroke	3 (11.5)	3 (15.0)	0 (0)	
T2DM	8 (30.8)	7 (35.0)	1 (16.7)	
CKD	1 (3.8)	1 (5.0)	0 (0)	
Cancer	1 (3.8)	1 (5.0)	0 (0)	
Psychiatric comorbidities				
Depression/Anxiety	13 (50.0)	10 (50.0)	3 (50.0)	
Schizophrenia	0 (0)	0 (0)	0 (0)	
Bipolar	1 (3.8)	1 (5.0)	0 (0)	
PTSD	1 (3.8)	1 (5.0)	0 (0)	
Insomnia	2 (7.7)	2 (10.0)	0 (0)	
Epilepsy	0 (0)	0 (0)	0 (0)	

[a]Kruskal-Wallis test; [b]Fisher's test. Abbreviations: IQR = interquartile range; COPD = chronic obstructive pulmonary disease; HTN = hypertension; HLD = hyperlipidemia; CHF = congestive heart failure; MI = myocardial infarction; T2DM = type 2 diabetes mellitus; CKD = chronic kidney disease; PTSD = posttraumatic stress disorder.

SUPPLEMENTARY APPENDIX A3: Smoking status and Cessation Aids Used in Trial (Per-protocol analysis)

	Total (N=26)	Intervention (N=20)	Control (N=6)	*p*-value
Fagerström score at baseline (Mean, SD)[a]	4.04 (1.73)	4.05 (1.7)	4 (2)	0.95
Fagerström score at baseline (N, %)[b]				
Low dependence	6 (23.1)	5 (25.0)	1 (16.7)	0.87
Low to moderate dependence	11 (42.3)	8 (40.0)	3 (50.0)	
Moderate dependence	9 (34.6)	7 (35.0)	2 (33.3)	
High dependence	0 (0)	0 (0)	0 (0)	
What was prescribed (N, %)[c]				
NRT	12 (48.0)	9 (45.0)	3 (60.0)	0.64
Chantix or Bupropion	13 (52.0)	11 (55.0)	2 (40.0)	
Missing	1	0	1	
CO level (Median, IQR)				
Baseline[b]	24.5 (17-35)	31 (18.5-36.5)	19 (15-24)	0.27
1 month[b]	3 (2-5)	3 (2-5)	1.5 (1-2)	0.04
3 months[b]	3 (2-10)	4 (3-12)	1.5 (1-2)	0.06

[a]t-test; [b]Kruskal-Wallis test; [c]Fisher's test; Abbreviations: SD = standard deviation; NRT = nicotine replacement therapy; CO = carbon monoxide.

SUPPLEMENTARY APPENDIX A4: Quit Rate at 1 month and 3 months (Per-protocol analysis)

Measure of Quit Rate	Total (N=26)	Intervention (N=20)	Control (N=6)	*p*-value[a]
**Quit Rate at 1 Month**				
Current smoker (N, %)				
Yes	3 (11.5)	3 (15.0)	0 (0)	0.99
No	23 (88.5)	17 (85.0)	6 (100)	
Smoked in past 7 days (N, %)				
Yes	3 (11.5)	3 (15.0)	0 (0)	0.99
No	23 (88.5)	17 (85.0)	6 (100)	
CO smoking status (N, %)				
Current smoker	4 (15.4)	3 (15.0)	1 (16.7)	0.99
Not smoking	22 (84.6)	17 (85.0)	5 (83.3)	
CO smoking status + self-report (N, %)				
Still smoking	4 (15.4)	3 (15.0)	1 (16.7)	0.99
Not smoking	22 (84.6)	17 (85.0)	5 (83.3)	
**Quit Rate at 3 Months**				
Current smoker (N, %)				

Yes	6 (23.1)	6 (30.0)	0 (0)	0.28
No	20 (76.9)	14 (70.0)	6 (100)	
Smoked in past 7 days (N, %)				
Yes	6 (23.1)	6 (30.0)	0 (0)	0.28
No	20 (76.9)	14 (70.0)	6 (100)	
CO smoking status (N, %)				
Current smoker	7 (26.9)	6 (30.0)	1 (16.7)	0.99
Not smoking	19 (73.1)	14 (70.0)	5 (83.3)	
CO smoking status + self-report (N, %)				
Still smoking	9 (34.6)	8 (40.0)	1 (16.7)	0.38
Not smoking	17 (65.4)	12 (60.0)	5 (83.3)	

[a]Fisher's test; Abbreviations: CO = carbon monoxide.

SUPPLEMENTARY APPENDIX B: Physical and Psychiatric Comorbidities among Study Participants

	Total (N=111)	Intervention (N=85)	Control (N=26)	*p*-value
Number of comorbidities (Median, IQR)				
Overall[a]	3 (2-4)	3 (2-4)	3 (1-4)	0.53
Physical[a]	2 (1-3)	2 (1-3)	2 (1-3)	0.93
Psychiatric[a]	1 (0-2)	1 (0-2)	1 (0-1)	0.31
Number of comorbidities (N,%)[c]				

<3 comorbidities	46 (41.4)	34 (40.0)	12 (46.2)	0.58
≥3 comorbidities	65 (58.6)	51 (60.0)	14 (53.8)	
≥1 comorbidity (N, %)				
Overall[b]	102 (91.9)	80 (94.1)	22 (84.6)	0.20
Physical[c]	88 (79.3)	69 (81.2)	19 (73.1)	0.49
Psychiatric[c]	72 (64.9)	56 (65.9)	16 (61.5)	0.86
Physical comorbidities (N, %)				
COPD[c]	26 (23.4)	21 (24.7)	5 (19.2)	0.61
Asthma[b]	9 (8.1)	6 (7.1)	3 (11.5)	0.42
HTN[c]	64 (57.7)	46 (54.1)	18 (69.2)	0.11
HLD[c]	46 (41.4)	36 (42.4)	10 (38.5)	0.83
CHF[b]	7 (6.3)	6 (7.1)	1 (3.8)	0.99
MI/stroke[b]	16 (14.4)	13 (15.3)	3 (11.5)	0.99
T2DM[c]	38 (34.2)	30 (35.3)	8 (30.8)	0.76
CKD[b]	8 (7.2)	7 (8.2)	1 (3.8)	0.68
Cancer[b]	9 (8.1)	6 (7.1)	3 (11.5)	0.42
Psychiatric comorbidities (N, %)				
Depression/anxiety[c]	65 (58.5)	51 (60)	14 (53.8)	0.72
Schizophrenia[b]	7 (6.3)	6 (7.1)	1 (3.8)	0.99
Bipolar[b]	17 (15.3)	14 (16.5)	3 (11.5)	0.76
PTSD[b]	7 (6.3)	6 (7.1)	1 (3.8)	0.99
Insomnia[b]	19 (17.1)	15 (17.6)	4 (15.4)	0.99

Epilepsy[b]	6 (5.4)	5 (5.9)	1 (3.8)	0.99

[a]Kruskal-Wallis test; [b]Fisher's test; [c]Chi-square test; Abbreviations: IQR = interquartile range; COPD = chronic obstructive pulmonary disease, HTN = hypertension; HLD = hyperlipidemia; CHF = congestive heart failure; MI = myocardial infarction; T2DM = type 2 diabetes mellitus; CKD = chronic kidney disease; PTSD = posttraumatic stress disorder.

© 2019 Evoy K.E.
